# Through-Wall Multiple Targets Vital Signs Tracking Based on VMD Algorithm

**DOI:** 10.3390/s16081293

**Published:** 2016-08-15

**Authors:** Jiaming Yan, Hong Hong, Heng Zhao, Yusheng Li, Chen Gu, Xiaohua Zhu

**Affiliations:** School of Electronic and Optical Engineering, Nanjing University of Science and Technology, Nanjing 210094, China; jamlff2494@163.com (J.Y.); hongnju@njust.edu.cn (H.H.); soniczhao@live.com (H.Z.); leassun@126.com (Y.L.); gc_njust@163.com (C.G.)

**Keywords:** ultra-wideband radar, variational mode decomposition, time-frequency analysis, through-wall detection

## Abstract

Targets located at the same distance are easily neglected in most through-wall multiple targets detecting applications which use the single-input single-output (SISO) ultra-wideband (UWB) radar system. In this paper, a novel multiple targets vital signs tracking algorithm for through-wall detection using SISO UWB radar has been proposed. Taking advantage of the high-resolution decomposition of the Variational Mode Decomposition (VMD) based algorithm, the respiration signals of different targets can be decomposed into different sub-signals, and then, we can track the time-varying respiration signals accurately when human targets located in the same distance. Intensive evaluation has been conducted to show the effectiveness of our scheme with a 0.15 m thick concrete brick wall. Constant, piecewise-constant and time-varying vital signs could be separated and tracked successfully with the proposed VMD based algorithm for two targets, even up to three targets. For the multiple targets’ vital signs tracking issues like urban search and rescue missions, our algorithm has superior capability in most detection applications.

## 1. Introduction

Through-wall detection has become a fundamental topic in homeland security, rescue missions and obscured objects detecting applications in recent years [1,2,3,4,5,6,7,8]. The traditional optical image sensors fail to provide visual information behind walls and shelters. The through-wall radar sensors, especially the ultra-wideband (UWB) radar have therefore become the preferred choices. Different from other radar systems, UWB radar system has strong penetrating power compare to the optical system, and has high range resolution and good resolving ability compare to the continuous wave (CW) radar system [9,10,11,12,13,14]. By using impulse signal or frequency modulated (FM) signal, UWB radar can not only measure the micro-doppler movements but can also distinguish the closely positioned targets.

Under this circumstance, through-wall human detection using UWB radar has emerged as one of the keen research topic. The applications of UWB system include indoor target localization, target tracking and multi-dimensional targets imaging [15,16,17,18]. In the meantime, with the development of remote biomedical monitoring, Doppler radar detection of vital signs has become a promising technique for health monitoring and life sensing applications [19,20,21,22,23]. This technique is based on the detection of significant features of human periodic motions, and UWB radar is a great candidate to receive signals containing periodic motions information from human targets related to the periodic contraction of lungs and the beating of hearts in the process of breathing and heartbeat respectively.

According to the degree of difficulty of the detection, the through-wall vital signs detection can divide into two parts, single target detection and multiple targets detection. For single target detection, a great deal of work has been achieved in the field of detecting human respiration, heartbeats and gait [24,25,26]. Compared with the various work of detecting a single target, the multiple targets detection faces difficulties like low resolution and overlapped harmonics of respiration waveform. Only a few effective algorithms have been proposed to detect human respiration through a wall [27,28,29]. In [27], a self-injection-locked (SIL) radar was presented for ranging and vital signs detection of targets through wooden wall when it worked in frequency modulated continuous wave (FMCW) mode. However, the FMCW transmitter signal can easily leak and susceptible to near ground clutter. In [28], the impulse UWB radar system was successfully used to monitor the micro-Doppler signatures like breathing rates of two subjects through a cement wall. In [29], the group used UWB radar system to detect and identify life characteristics of two targets at different distance. However, the number of multiple targets in these cases is limited to two person and they cannot track the rapid change of the respiration signals. Therefore, the through-wall detection of vital signs features from multiple targets located in the same distance is easily to be neglected.

In this paper, we apply a SISO UWB radar system with high precision in through-wall multiple targets vital signs detection. Based on the system, we addressed VMD algorithm [30] to decompose the respiration characteristics from human targets in the same distance. Then we used Hilbert transform to track the time-varying respiration signals. The UWB system has high resolution and could provide great penetration. So it could provide high accuracy and separation in through-wall multiple targets vital signs detecting applications. The VMD algorithm has not been applied to radar signal processing before and it’s a novel time domain algorithm to separate different frequency signals in low noise levels. The method has been experimentally validated in this paper and has the ability to detect multiple targets respiration rates through the wall.

This paper is organized as follows. In Section 2, we describe the theory of UWB radar vital signs measurement. In Section 3, the multiple targets vital signs tracking algorithm is described. In Section 4, UWB radar system and its configuration are proposed, and the experimental results are used to assess the effectiveness of the proposed procedure. Finally, Section 5 contains the conclusions.

## 2. Theory of UWB Radar Vital Signs Measurement

**Figure 1:**
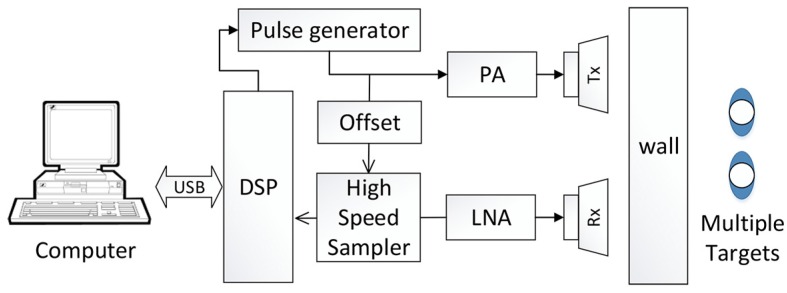
Block diagram of the single-input single-output (SISO) ultra-wideband (UWB) radar system.

The UWB impulse radar is functioning based on the phase estimation of the signal reflected by multiple targets. The block diagram of SISO UWB radar system is illustrated in Figure 1.

We assume that targets are located at a fixed distance $d_{0}$ with multiple targets physiological displacement x(t). Thus, the total distance between the transmitter and targets d(t) can be expressed as:

(1) $$d\left(t\right)=d_{0}+x\left(t\right)$$

where x(t) can be written as:

(2) $$x\left(t\right)=\sum_{i=1}^{m}a_{i}\sin\left(2\pi f_{i}t\right)$$

where i=[1,2,⋯,m] denotes the number of the targets, $a_{i}$ and $f_{i}$ represent the respiration amplitude and frequency of the *i*-th target, respectively.

In this situation, the received signal can be represented as the sum of the responses of the channel, So the impulse responses h(t,τ) in the range profile reflected from multiple targets will be:

(3) $$h\left(t,\tau \right)=h_{c}\left(t,\tau \right)+\sum_{i=1}^{m}A_{i}\partial \left(\tau -\tau_{iv}\left(t\right)\right)$$

$h_{c}\left(t,\tau \right)$ denotes the response from environmental noise and other multipath component. Where $\tau_{iv}\left(t\right)$ is the time delay and $A_{i}$ is the amplitude of the pulse reflected on the *i*-th target. This indicates that the respiration movements modulate the received signal.

Due to stationarity of reflectors in the scene, each time delay $\tau_{v}$ associated with the respirations are modeled as the sum of time of arrival $\tau_{0}$ plus some sinusoidal delays associated to the respiration displacements of multiple targets:

(4) $$\tau_{v}\left(t\right)=\frac{2d(t)}{C}=\tau_{0}+\sum_{i=1}^{m}\tau_{i}\sin\left(2\pi f_{i}t\right)$$

where *C* denotes the propagation speed, and $\tau_{i}$ represents the respiration displacement of *i*-th target. As the transmit signal T(t) is reflected by the targets, the received signal r(t,τ) can be written as:

(5) r(t,τ)=T(t)×h(t,τ)

Then discrete instants the received waveforms r(t,τ) and the values are stored in a J×N matrix R=R[J,N]:

(6) $$R\left[J,N\right]=r\left(t=jT_{s},\tau =nT_{f}\right)$$

where j=[1,2,⋯,J] indicates the slow time index and n=[1,2,⋯,N] indicates the fast time index. $T_{s}$ and $T_{f}$ are the sampling period in slow time and fast time, respectively.

## 3. Multiple Targets Vital Signs Tracking Algorithm

**Figure 2:**
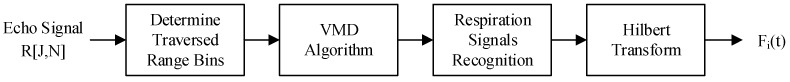
Block diagram of the multiple targets vital signs tracking algorithm.

In this section, our algorithm has been used to obtain the through-wall multiple targets vital signs signals. A general block diagram of the algorithm is shown in Figure 2. The detail steps for implementing the algorithm are as follows:

### 3.1. Determine Traversed Range Bins

Through-wall multiple targets detection usually can be divided into two cases: same distance and different distances. This section is placed at finding out which range bin contains the most intense chest movement signal. If targets are located in different range bins, it’s easy to detect and separate each target based on UWB impulse system. So we are particularly interested in the condition with the same range bin. The first step is to select the candidate range bin which involves desired respiration signals.

The received signal R[J,N] derived from the UWB radar system divide into *J* range bins and *N* discrete-time sequences. We apply a moving window of 2048 points to track the candidate range bin based on calculate the variance $V_{j}$, which can be described by:

(7) $$V_{j}={\left(x_{j,1}-\bar{x_{j}}\right)}^{2}+\cdots +{\left(x_{j,n}-\bar{x_{j}}\right)}^{2}+{\left(x_{j,N}-\bar{x_{j}}\right)}^{2}$$

where $\bar{x_{j}}$ is the average value of *J* range bins and $x_{j,n}$ is the *n*-th slow time sample of the *j*-th range bin.

After calculating the variance of each pulse in each range bin and find the range bin with the maximum variance, the candidate range bin *V* can be picked to get the desired respiration signals. The expression can be written as:

(8) $$V=\max V_{j}$$

Here we get the original signal $f_{v}\left(t\right)$ from range bin *V*, which can be used later on for respiration signals separation. After $f_{v}\left(t\right)$ is obtained and then, is filtered by a low-pass filter from 0 Hz to 0.7 Hz (0.7 Hz corresponding to 42 Beats/Minute) to reduce the magnitude of the high frequency component.

### 3.2. Respiration Signals Separation with the VMD Algorithm

VMD algorithm is an entirely non-recursive variational mode decomposition method, and the modes are extracted concurrently. The recovered VMD modes constitute a nice partition of the input spectrum, with each mode being clearly dominant around the signal, and it is capable to captures the relevant center frequencies quite precisely [30]. This algorithm is much more robust to sampling and noise. In this paper, we use VMD algorithm to achieve our multiple targets vital signs tracking algorithm.

First of all, mirror-symmetric extension the preprocessed signal $f_{v}\left(t\right)$ and obtain the echo signal f(t) , and the second step is updates the modes $\mu_{k}\left(t\right)$ and the center frequencies $\omega_{k}$, which *K* is the number of modes to be recovered. We can get the updates $\mu_{k}\left(t\right)$ in the *n*-th cycle using the following formulas:

(9) $${\hat{\mu }}_{k}^{n+1}=\frac{\hat{f}\left(\omega \right)-\sum_{i\neq k}{\hat{\mu }}_{i}\left(\omega \right)+\frac{\hat{\lambda }\left(\omega \right)}{2}}{1+2\alpha {\left(\omega -\omega_{k}\right)}^{2}}$$

where $\hat{f}\left(\omega \right)$ is the Fourier transform of the echo signal f(t), $\hat{\mu_{i}}\left(\omega \right)$ is the Fourier transform of *i*-th sub-signal $\mu_{i}\left(t\right)$ and *α* represents the balancing parameter of the data-fidelity constraint. Which is clearly identified as a wiener filtering of the current residual, with signal prior $1/{\left(\omega -\omega_{k}\right)}^{2}$ . To update $\omega_{k}$ is formulated by:

(10) $$\omega_{k}^{n+1}=\frac{\int_{0}^{\infty }\omega \left|{\left.{\hat{\mu }}_{k}\left(\omega \right)\right|}^{2}d\omega \right.}{\int_{0}^{\infty }\left|{\left.{\hat{\mu }}_{k}\left(\omega \right)\right|}^{2}d\omega \right.}$$

where ${\hat{\mu }}_{k}\left(\omega \right)$ is the Fourier transform of the sub-signal $\mu_{k}\left(t\right)$. Dual ascent for all ω≥0. In VMD algorithm, the Lagrangian multiplier *λ* can be used to enforce exact reconstruction of the input signal [30], the updates of Lagrangian multipliers *λ* is as follows:

(11) $${\hat{\lambda }}^{n+1}\left(\omega \right)\leftarrow {\hat{\lambda }}^{n}\left(\omega \right)+\tau \left(\hat{f}\left(\omega \right)-\sum_{k}{\hat{\mu }}_{k}^{n+1}\left(\omega \right)\right)$$

where *τ* is the update parameter of Lagrangian multiplier (pick 0 for denoising), and the cut-off condition of $\mu_{k}$ is as follows:

(12) $$\frac{\sum_{k}{∥{\hat{\mu }}_{k}^{n+1}-{\hat{\mu }}_{k}^{n}∥}_{2}^{2}}{{∥{\hat{\mu }}_{k}^{n}∥}_{2}^{2}}<\epsilon$$

where *ϵ* is the tolerance of convergence criterion (typically around 10^−6^). The final step is reconstruction each mode $\mu_{k}\left(\omega \right)$ converges after iterations. Then recognize the respiration signals and obtain $K_{R}$ modes time domain respiration signals $\mu_{K_{R}}\left(t\right)$ and the each center frequency $\omega_{K_{R}}$.

### 3.3. Calculation of Instantaneous Frequency

Instantaneous frequency ${\hat{f}}_{i}\left(t\right)$ is calculated via Hilbert transform [31] from $\mu_{i}\left(t\right)$, $i=[1,2,\cdots ,K_{R}]$. To eliminate the possible random fluctuation induced by noise and computational errors, these instantaneous frequencies are smoothed by a simple averaging after a window function,

(13) $$F_{i}\left(t\right)=\frac{1}{T}\int_{-2/T}^{+2/T}{\hat{f}}_{i}\left(\tau \right)W\left(\tau -t\right)d\tau ,\left(i=1,2,3,\cdots \right)$$

where W(t) is a rectangular function of width *T* and height 1, with T set to be three times of the dominant oscillatory period of the selected modes.

Considering the impact of a real experimental environment, we carried out a comparative simulation experiments to verify the performance of our algorithm. The simulated signal is a composition of two simple components which simulated respiration rates were set at 12 Beats/Minute (0.2 Hz) and 21 Beats/Minute (0.35 Hz) respectively. To verify the anti-noise performance of our algorithm, we compared the simulated results between noiseless condition and one with SNR of −10 dB. The comparison results are as follows.

**Figure 3:**
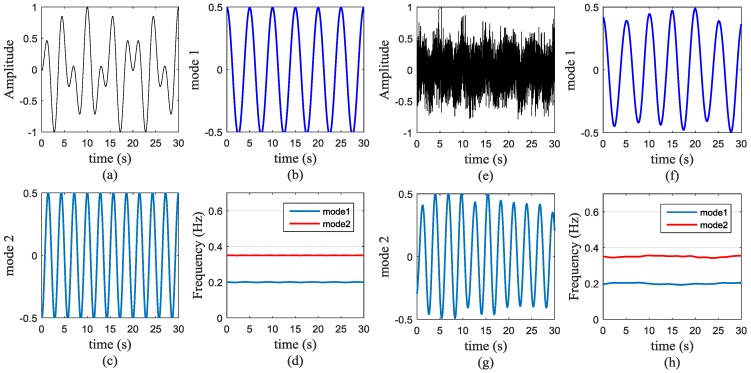
Simulated respiration signals and separation results. (**a**) Simulated signal, (**b**) and (**c**) two modes decompose by Variational Mode Decomposition (VMD) based algorithm; (**d**) time-varying respiration signals; (**e**) Simulated signal with −10 dB SNR, (**f**) and (**g**) two modes decompose by VMD based algorithm; (**h**) time-varying respiration signals.

Figure 3a shows the simulated signal and the two respiration signals decompose by VMD algorithm are shown in Figure 3b,c. Then the results from Hilbert transform after VMD algorithm is presented in Figure 3d. From this picture, our algorithm not only gets the accurate frequency information but also separates each respiratory signal dynamically. At the control group, we add white Gaussian noise and change the SNR into −10 dB to simulate the experiment. Figure 3e–h correspond to the former four figures respectively. For sufficiently low noise levels, we calculate the correlation of each mode. The correlation of Figure 3b,f is 97.66%, the correlation of Figure 3c,g is 95.66%. The results show the VMD based algorithm have a good anti-noise performance.

## 4. UWB Radar System and Experimental results

The block diagram of the SISO UWB radar system is illustrated in Figure 1. The main control unit is a NVA6100 chip CMOS impulse radar system. The radar chip has a high accuracy with a range equivalent sampling rate of 4 mm and a simultaneous observation of 512 depths. We use two vivaldi antenna and the antenna gain is 6 dBi. The system can be divided into two sections: the transmitter and the receiver.

**Figure 4:**
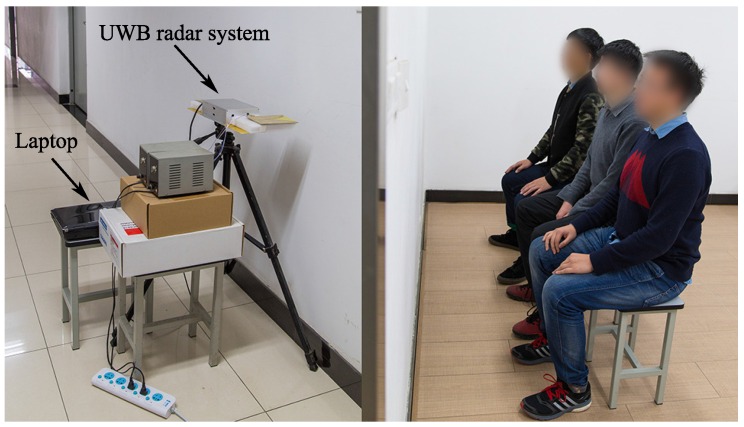
Experimental environment.

In the transmitter, the pulse generator is responsible for the signal generation. It generates the first-order Gaussian signal. The bandwidth is from 0.85 GHz to 9.55 GHz (−10 dB) and the pulse repetition frequency (PRF) is 48 MHz. Then the signal is amplified by the power amplifier and emitted by transmitting antenna. The maximum transmitter power is increased from −19 dBm to 1 dBm. In the receiver, the reflected signal is firstly received by the receiving antenna. After a low-noise amplifier (LNA), the analog signal is sampled by the high-speed sampler whose fast time sampling frequency is 39 GHz [32]. Then the digital signal is sent to the DSP. After slow time sampling which frequency is 152.6 Hz, the signal is transferred to a desktop through a universal serial bus (USB) for subsequent processing. The UWB radar system is presented in Figure 4.

Based on the system, we designed four different types of experiments to track vital signs features from multiple targets through the wall. In each experiment, we set each target almost at the same distance from the antenna and make sure the environment variables virtually unchanged. The experimental settings and results are as follows.

### 4.1. Two Targets with Constant Respiration Rates

**Figure 5:**
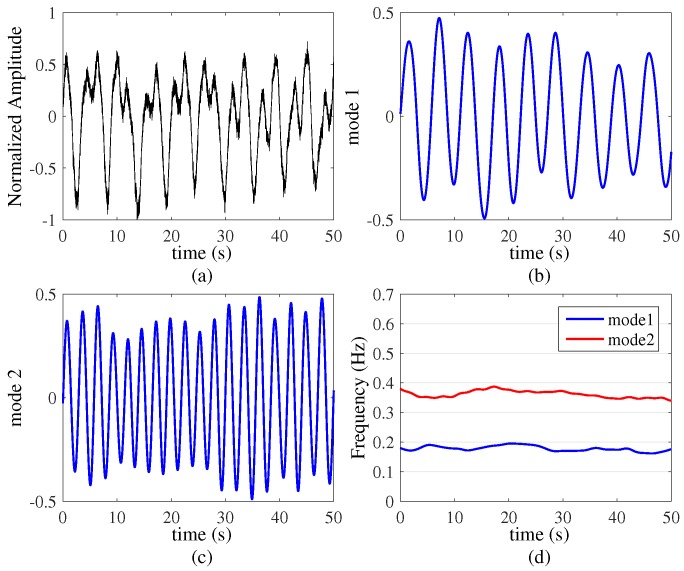
Two targets with constant respiration rates (k=2, α=20,000). (**a**) Original signal; (**b**) and (**c**) are two modes decompose by VMD based algorithm; (**d**) is the time-varying respiration signal.

In the first set of experiments, we reproduced the simulation signals by using the real respiration signals. The UWB radar system was placed at 0.2 m on one side of the wall. Two human targets were located behind a 0.15 m thick concrete brick wall and they were both positioned on the other side of the wall at a standoff distance (i.e., the distance between the targets and the wall) of 1.5 m. They kept constant respiration rates for about 50 s. The results are shown in Figure 5.

The original signal is shown in Figure 5a, and the VMD based algorithm results are shown in Figure 5b,c, which contain two objects behind the wall. Because their chest has different vibration amplitudes, we can clearly see the two waveforms out-of-phase and our algorithm obtains the respiration dynamic characteristics of two targets. Then in Figure 5d, the time-frequency analysis results by Hilbert transform show exhibiting respiration rates of 0.2 Hz and 0.37 Hz, which are 12 Beats/Minute and 22 Beats/Minute respectively. From the picture, we can see the two targets remain stationary and smooth respiratory rates clearly in 50 s by using our algorithm, and do not show any interference from the wall. On the basis of the results of this experiment, we performed the experiment three times and the results show excellent separating performance.

### 4.2. Two Targets with Piecewise-Constant Respiration Rates

To demonstrate the effectiveness of VMD based algorithm, another experiment was performed in which two human targets (target A and target B) with different respiration time periods and same distance were imaged. The two targets were situated on the same distance, where the targets were positioned 2 m from the UWB radar system. First of all, target A maintained steady respiration for about 25 s, in the meantime, target B held his breath for about 25 s. When the time node arrived, target A switched to hold his breath and target B converted into deep breathing for another 25 s till the end of the experiment. We assume that if the amplitude of the signal is less than 25% of the normal respiration signals, it is not a effective signal and we smooth the periods by using a three times dominant oscillatory period of the selected modes. This experiment still lasted about 50 s.

**Figure 6:**
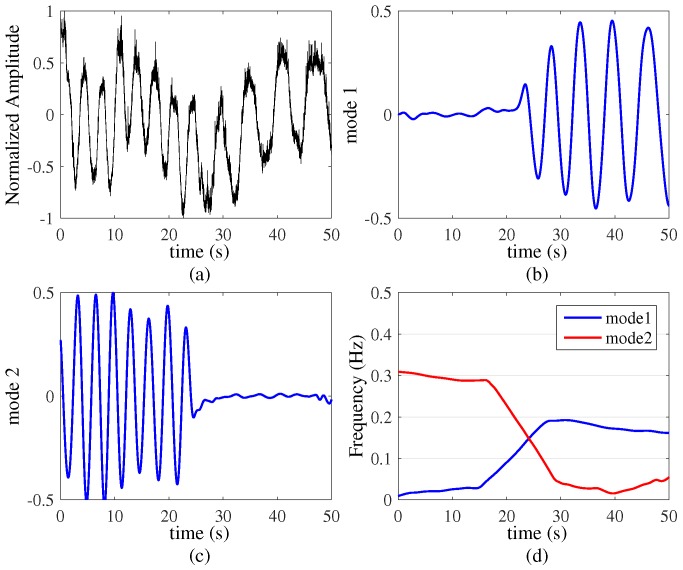
Two targets with piecewise-constant respiration rates (k=3, α=20,000). (**a**) Original signals; (**b**) and (**c**) two modes decompose by VMD based algorithm; (**d**) time-varying respiration signal.

**Figure 7:**
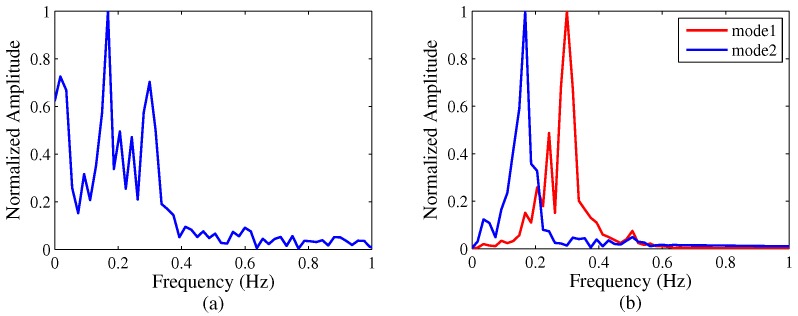
The comparison of the traditional FFT method and VMD based method. (**a**) FFT results without VMD based algorithm; (**b**) FFT results with VMD based algorithm.

The UWB echo data are shown in Figure 6a. From this figure, it is difficult to find a respiration tendency from the two targets. Then the respiration signals separation results are shown in Figure 6b,c and we can see the dynamic characteristic of the two respiration modes clearly. Because target B held his breath first, he has a large period of deep breathing until the end of the experiment. Then in Figure 6d, the time-frequency analysis results indicate a respiration rate of 20 Beats/Minute for the seated target A, and a respiration rate of 12 Beats/Minute for the seated target B, respectively. Finally, Figure 7a shows the FFT results without VMD based algorithm and Figure 7b shows the FFT results with VMD based algorithm, which the comparison is very obvious. The VMD based algorithm removes the interference of the wall and separates the respiration signal of each target very well.

### 4.3. Two Targets with Time-Varying Respiration Rates

To further demonstrate the high-resolution decomposition of our method, the third experiment was performed a more difficult environment in which two human targets with time-varying respiration rates and in the same distance (The position is 2 m from the UWB radar system). We controlled the respiration rate of target A, which increased from 12 Beats/Minute (0.2 Hz) to 18 Beats/Minute (0.3 Hz) in 30 s. In the meantime, target B’s respiration rate varied from 30 Beats/Minute (0.5 Hz) to 24 Beats/Minute (0.4 Hz).

**Figure 8:**
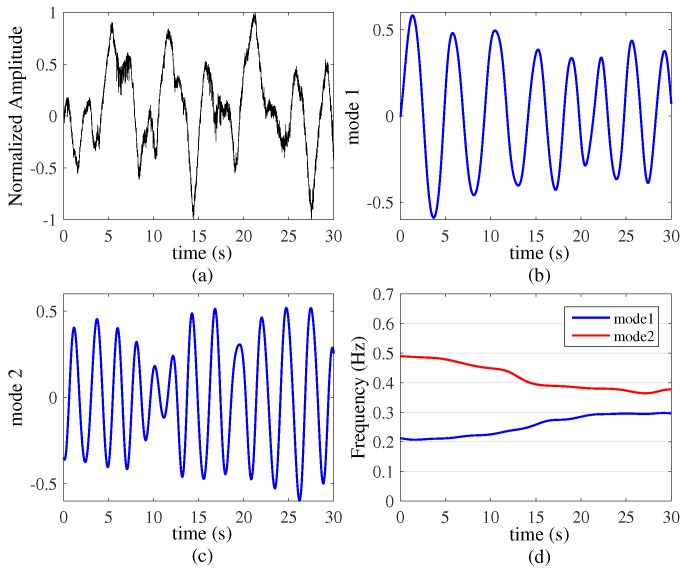
Two targets with time-varying respiration rates and VMD based algorithm results (k=3, α=20,000). (**a**) Original signals; (**b**) and (**c**) two modes decompose by VMD based algorithm; (**d**) time-varying respiration signals.

The UWB echo data are shown in Figure 8a, from this figure, it is difficult to get the time-varying respiration rate from each target by using the FFT algorithm directly. Then the VMD based algorithm results are shown in Figure 8b,c, from which we can see the dynamic characteristics of the two respiration signals clearly. Finally in Figure 8d, from the Hilbert transform we are able to see two instantaneous respiration rates more directly. Target A varied from 12 Beats/Minute (0.2 Hz) to 18 Beats/Minute (0.3 Hz) and target B’s respiration rate varied from 30 Beats/Minute (0.5 Hz) to 24 Beats/Minute respectively. The results show our algorithm has the excellent capability to detect multiple targets time-varying respiration signals.

### 4.4. Three Targets with Constant Respiration Rates

**Figure 9:**
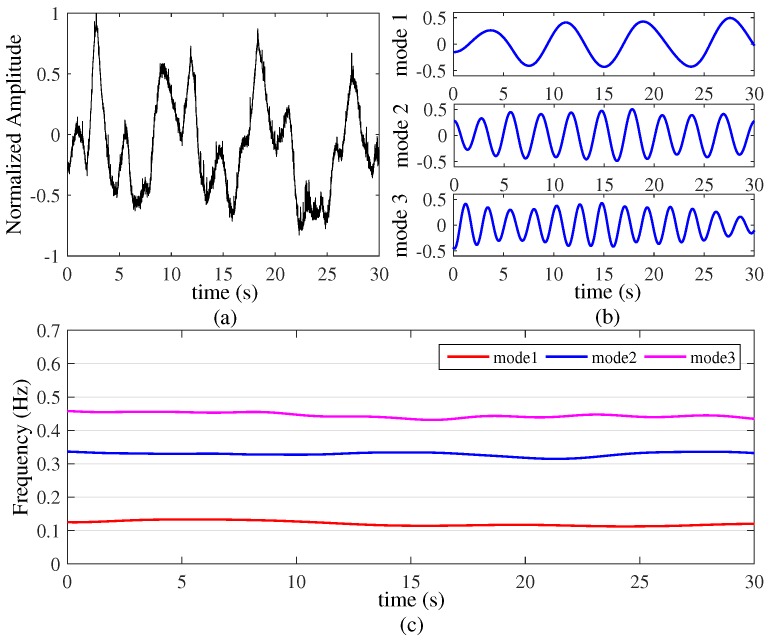
Three targets with constant respiration rates (k=4, α=20,000). (**a**) Original signals; (**b**) three modes decompose by VMD based algorithm; (**c**) time-varying respiration signal.

Finally, the final experiment was carried out to localize and detect the respiration of three stationary human targets (targets A–C). The three human targets with constant respiration rates are located in the same distance from the UWB radar. The distance from their chests to the wall is 1.5 m. Everyone maintains a constant respiration rate and this experiment lasted about 30 s. We performed the experiment three times and the results were 100% successful. The result shown in Figure 9.

The original data are presented in Figure 9a and the three sub-signals are illustrated in Figure 9b. From the picture, we can see the dynamic characteristic with different periods from three targets. The respiration characteristics of three targets are presented in the Figure 9c, which shows that target A has a respiration rate of 0.12 Hz (7 Beats/Minute), target B has a respiration rate of 0.34 Hz (20 Beats/Minute) and target C has a respiration rate of 0.45 Hz (27 Beats/Minute) respectively. Based on the results of this experiment, we still performed the experiment three times and the separating results were all successful. This experiment demonstrates the VMD based algorithm we proposed has high accuracy and strong decomposition to detect the constant vital signs signals of three targets.

## 5. Conclusions

In this paper, we consider the superimposition and interference issues of echo signals from the same distance, which most through-wall multiple targets detecting applications are unable to detect. The modes decomposition method, i.e., the VMD algorithm, has been elegantly extended to SISO UWB radar system. A proof of concept is provided by using experimental data collected in a laboratory environment with up to three targets at different respiratory status, i.e., constant, piecewise-constant and time-varying respiration rate. The proposed technique has proven to provide significant advantages in dynamic monitoring while maintaining desirable signal quality. The results show that the VMD based algorithm is capable of detecting time-varying respiration rate of each target in the same distance. In the near future, we will address more complex experiments, such as detecting heartbeats and tracking through-wall multiple human respiration rates.
